# Prevalence of simian malaria among macaques in Malaysia (2000–2021): A systematic review

**DOI:** 10.1371/journal.pntd.0010527

**Published:** 2022-07-18

**Authors:** Janeeca Sam, Nadia Aqilla Shamsusah, Amatul Hamizah Ali, Rozita Hod, Mohd Rohaizat Hassan, Hani Kartini Agustar

**Affiliations:** 1 Department of Bioscience and Biotechnology, Faculty of Science & Technology, Universiti Kebangsaan Malaysia, Bangi, Selangor, Malaysia; 2 Department of Earth Sciences and Environment, Faculty of Science & Technology, Universiti Kebangsaan Malaysia, Bangi, Selangor, Malaysia; 3 Department of Chemical Sciences, Faculty of Science & Technology, Universiti Kebangsaan Malaysia, Bangi, Selangor, Malaysia; 4 Department of Community Health, Faculty of Medicine, Universiti Kebangsaan Malaysia, Kuala Lumpur, Malaysia; Universidade Federal de Minas Gerais, BRAZIL

## Abstract

**Background:**

The aim of Malaysia to eliminate malaria nationwide by 2020 seems need to be prolonged. Whilst Malaysia has successfully eliminated human malaria transmission, simian malaria parasites such as *Plasmodium knowlesi*, *P*. *cynomolgi*, *P*. *inui* and *P*. *cynomolgi* are the emerging cause of malaria in humans. The epidemiological study of simian malaria in primates provides useful information in identifying the risk of human-macaques *Plasmodium* infection.

**Methodology/Principal findings:**

This study was performed to gather all available data in terms of simian malaria epidemiology study among macaques in Malaysia over the last two decades. This systematic review was conducted according to the PRISMA guidelines to select appropriate articles as references. Data searches were performed through international databases such as Google Scholar, PubMed, CrossRef, Scopus, Web of Science and Science Direct for original articles published from 2000 until 2021. The review identified seven simian malaria epidemiology studies in Malaysia over the 20-year study period. Most studies were conducted in Peninsular Malaysia (5/7; 71%) followed by East Malaysia (2/7; 29%). All studies showed positive detection of *Plasmodium* parasites in macaques. The most prevalent *Plasmodium* species in macaques was *P*. *inui* (49.27%) and the least prevalent was *P*. *fieldi* (4.76%). The prevalence of simian malaria was higher in East Malaysia compared to Peninsular Malaysia. The mono, dual and triple infection types were the most common among macaques.

**Conclusion/Significance:**

The non-human primates like macaques are the reservoir of simian plasmodium in Malaysia. Hence, the study of host epidemiology is an important insight to public health management as there is a high occurrence of simian malaria in Malaysia. The right measurement can be taken as well to prevent the transmission of simian malaria from macaques to humans.

## Author summary

Macaques are the most abundant primates in south east Asia including Malaysia. Due to deforestation, macaques came closer to human settlements searching for food. Macaques like the long-tailed and pig-tailed harbouring several *Plasmodium* species that can cause zoonotic malaria in humans. Close contact of human and macaques cause zoonotic transmission of simian malaria. The simian plasmodium such as *P*. *knowlesi*, *P*. *inui* and *P*. *cynomolgi* have been found infecting humans in Malaysia; mainly in East Malaysia (Borneo). Zoonotic malaria poses great risk to public health as prolonged in treatment often lead to fatal outcomes. Hence the knowledge of prevalence and diversity is important to access, this can therefore enlighten the authorities to plan a control strategy that will minimize the zoonotic transmission between non-human primate host to human. This systematic review has summarised all publish data of macaques-plasmodium infection from the year 2000–2021 by using PRISMA guidelines. Our result showed that *P*. *inui* (49.27%) is the most prevalent *Plasmodium* species found in macaques, followed by *P*. *cynomolgi* (33.05%) and *P*. *knowlesi* (26.86%). Simian plasmodium prevalent was also found higher in East Malaysia (97.0%) compared to Peninsular Malaysia (45.18%). The significant increase of simian malaria incidences in human have jeopardized the national malaria elimination programme. Hence, this study provides a compact insight into the plasmodium epidemiology of macaques in Malaysia.

## Introduction

Malaria is one of the most prevalent vector-borne diseases around the world. Globally, there were approximately 229 million malaria cases and 409,000 deaths recorded in 2019 [1]. The hemoparasites in the genera *Plasmodium* are the causative agents of the malaria disease. Over the recent years, the burden of clinical malaria in Malaysia was predominantly due to infection by *P*. *knowlesi*, a malaria parasite of macaques. Many studies have shown that parasites are frequently transmitted from wild non-human primates (NHP) like long-tailed macaques (*Macaca fascicularis*) and pig-tailed macaques (*M*. *nemestrina*) or those in captivity to humans in a shared habitat [2]. Environmental changes such as deforestation and the associated exploitation of these new areas for agriculture has brought macaques and their vectors closer to human habitats [3]. Furthermore, the rising popularity of eco-tourism and recreational retreats such as hiking has also brought humans closer to primate’s natural habitat.

A wide range of primates including monkeys, apes and lemurs can be infected by *Plasmodium* [4]. There are at least 26 species of *Plasmodium* parasites that are capable of infecting non-human primates [5]. Seventeen species are prevalent in Asia [6] with *P*. *cynomolgi*, *P*. *inui* and *P*. *knowlesi* being potentially infectious to humans in the region [7].

In general, malaria parasites are host specific and cross-species transmission is less likely to happen. However, this perception changed after an impactful discovery in 2004 that a large focus of human infection with *P*. *knowlesi* was recorded in the Kapit District in Sarawak state, Malaysia [8]. Since then, *P*. *knowlesi* has been recognised as the fifth human malaria parasite along with the rest of four species: *P*. *malariae*, *P*. *vivax*, *P*. *ovale* and *P*. *falciparum*. Soon, human infections of *P*. *knowlesi* have since been reported throughout Southeast Asia [9,10]. *Plasmodium knowlesi* is primarily a simian malaria parasite that was first isolated from long-tailed macaques in the year 1932 [11]. In addition, the long-tailed macaques and pig-tailed macaques in particular, are reservoirs for *P*. *knowlesi*, *P*. *inui*, *P*. *cynomolgi*, *P*. *coatneyi* and *P*. *fieldi* [12]. As for simian malaria vectors, the *Anopheles* mosquitoes form Leucosphyrus group have been incriminated by early study [13,14] and recent studies have exhibited the *An*. *latens* as vector of knowlesi malaria in Kapit, Sarawak [15,16], *An*. *introlatus* in Hulu Selangor [17], and *An*. *cracens* from Dirus complex as the simian malaria vector in Kuala Lipis, Pahang [18,19]. More recent, *An*. *balabacensis* and *An*. *donaldi* have been incriminated as new vectors for simian malaria in Lawas, northern Sarawak [20]. Under laboratory conditions, *Anopheles* mosquitoes besides Leucosphyrus group has shown the ability to infect humans with *P*. *cynomolgi* together with *P*. *inui* through the bites [21–23].

The epidemiological surveys of *Plasmodium* infections in primates are needed to assess the risk of human exposure to zoonotic malaria [24]. The epidemiological study of *Plasmodium* in primates will also help in identifying the reservoir hosts and extend the understanding of the parasite evolutionary history. In Malaysia, several epidemiological studies have been conducted to assess the prevalence and diversity of *Plasmodium* infection among macaques. However, there is no detailed systematic review on the simian malaria prevalence in Malaysia over the past 20 years. Therefore, the aim of this study was to collect relevant published studies related to the prevalence of *Plasmodium* species in macaques and the prevalence of infection types through a systematic review from years 2000–2021.

## Methods

### Ethics statement

This is a systematic review and all the data recruited are publicly available. Therefore, ethical approval is not obtained.

### Search strategy

This systematic review was conducted using published studies on the detection and identification of *Plasmodium* among macaques in Malaysia. Eligible studies were identified in Google Scholar, PubMed, CrossRef, Scopus, Web of Science (Clarivate Analytics) and Science Direct (Elsevier) databases searched from the year 2000 to 2021. The search was commonly conducted using the search term [“Plasmodium” AND “Simian Malaria” AND “Malaysia”] of combination to obtain relevant articles. This systematic review was conducted according to the guidelines of PRISMA (Preferred Reporting Items for Systematic Reviews and Meta-analysis) [25].

### Eligibility criteria

The inclusion criteria for the article selection were: (a) the primary simian malaria research conducted in Malaysia; (b) the study subject were primates, specifically macaques; (c) the articles were published from the year 2000–2021; (d) full-text articles in English; (e) the articles must also provide a description of sample size, study site, *Plasmodium* diagnosis method, as well as the *Plasmodium* species and the number or percentage of infection.

For exclusion criteria, this study excludes review articles and articles that focused on: (a) human malaria; (b) simian malaria infection in human; (c) *Plasmodium* in vector; (d) *Plasmodium* genomic research; (e) diagnostic and antimalaria; (f) geographical and landscape.

### Data extraction

All searched articles were imported and combined into the Microsoft Excel 2019 software and duplicated files were removed. Based on the predetermined inclusion criteria, two independent review authors (SJ and NAS) determined qualified studies based on titles, language and abstracts. From the selected articles, relevant information such as name of the first author, year of publication, study region (Peninsular or East Malaysia), study state, period of sampling, study subject, sample size, analysis method for *Plasmodium* detection, the number/ percentage of *Plasmodium* positive infection as well as the type of simian malaria infection were extracted.

### Data analysis

Based on the extracted data, descriptive analysis was conducted using Microsoft Excel 2019 software. The total number of samples collected in each state of Malaysia was represented in map. The prevalence of *Plasmodium* species infection in macaques, type of simian malaria infection and simian malaria in different regions were calculated and tabulated.


$$Prevalence=\frac{Number\;of\;positive\;infections\;by\;Plasmodium\;sp.in\;macaques\;}{Total\;number\;of\;macaques\;}x\;100$$

## Results

### Data and study characteristics

**Fig 1:**
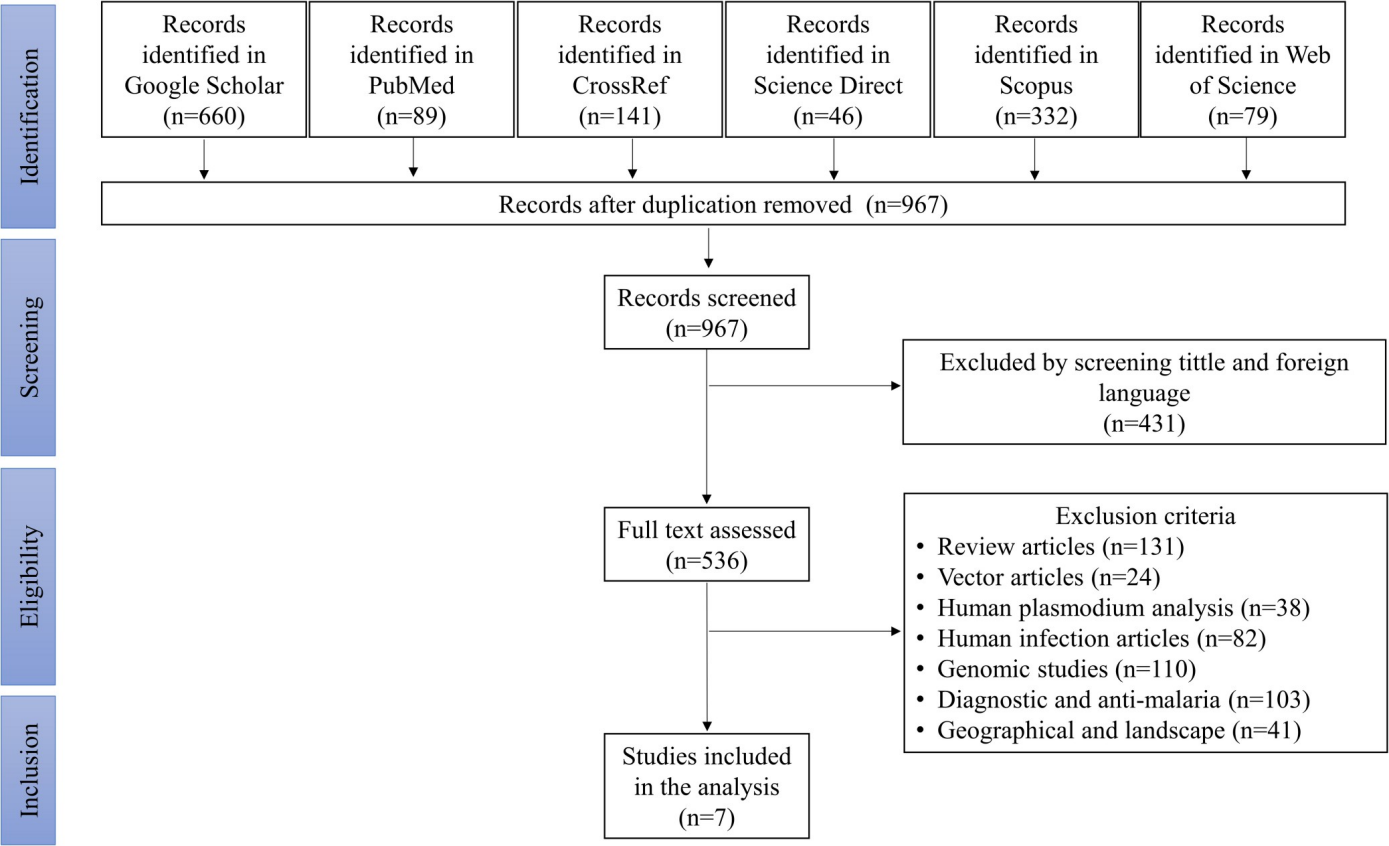
Flowchart of selected articles for the systematic review following the PRISM guidelines.

The literature search from six databases has generated 1347 results, which composed of 660 articles from Google Scholar, 89 from PubMed, 141 from CrossRef, 46 from Science Direct (Elsevier), 332 from Scopus and 79 from Web of Science. After duplicate articles have been removed, 967 articles were left for screening. After the screening of foreign languages, titles, and abstracts, 536 articles were left for further detailed evaluation (Fig 1). Most articles were excluded due to review articles and the studies of diagnostic and antimalaria properties. Other exclusion reasons include genomic studies, vector articles, human *Plasmodium* analysis, human infection articles and geographical and landscape articles. As a result, seven articles were selected in this study for data extraction [10,18,26–30] (Table 1).

**Table 1:** Summary of the main features of selected articles.

References	Study Area	Study State	Period of sample Collection	Study Subject	Sample Size, n	Analysis method	*Plasmodium* positive n (%)	*P*. *knowlesi* n (%)	*P*. *cynomolgi* n (%)	*P*. *coatneyi* n (%)	*P*. *feldi* n (%)	*P*. *inui* n (%)	Mono Infection n (%)	Dual infection n (%)	Triple Infection n (%)	Quadruple infection n (%)	Quintuple Infection n (%)
Vythilingam et al. (2008) [18]	Peninsular Malaysia	Pahang, KL, Selangor	2017	*1*. *Macaca fascicularis 2*. *Macaca nemestrina 3*. *Prebytis melalophos*	145	Nested PCR 18S rrna	75 (51.72%)	10 (13.33%)	-	-	-	-	-	-	-	-	-
Lee et al. (2011) [26]	East Malaysia	Kapit Sarawak	2004–2008	*1*. *Macaca fascicularis 2*. *Macaca nemestrina*	108	Nested PCR 18S rrna	101 (94%)	84 (83.16%)	60 (59.40%)	71 (70.29%)	4 (3.9%)	88 (87.12%)	10 (9.9%)	18 (17.82%)	30 (29.70%)	42 (41.58%)	1 (1%)
Akter et al. (2015) [10]	Peninsular Malaysia	Hulu Langat, KL	June 2014	*1*. *Macaca fascicularis*	70	Nested PCR 18S rrna	35 (50%)	21 (60%)	18 (51.4%)	16 (45.7%)	1 (2.9%)	23 (65.7%)	9 (25.7%)	11 (31.4%)	12 (34.3%)	3 (8.6%)	-
Muehlenbein et al. (2015) [27]	East Malaysia	Sabah	July 2010—November 2011	*1*. *Macaca fascicularis 2*. *Macaca nemestrina*	41	Nested PCR CytB gene	41 (100%)	6 (14.63%)	4 (9.75%)	2 (4.87%)	4 (9.75%)	17 (41.46%)	-	-	-	-	-
Huddin et al. (2019) [28]	Peninsular Malaysia	Kedah, Kelantan, KL, Pahang, Selangor, Terengganu	-	*1*. *Macaca fascicularis*	415	Nested PCR 18srrna gene	48 (11.6%)	48 (11.6%)	-	-	-	-	-	-	-	-	-
Amir et al. (2020) [29]	Peninsular Malaysia	Pahang, Perak, Johor	March-August 2016	*1*. *Macaca fascicularis 2*. *Macaca nemestrina*	103	Nestec PCR 18s rrna	64 (62.1%)	11 (10.68%)	42 (40.77%)	14 (13.59%)	4 (3.88%)	42 (40.77%)	31 (48.43%)	21 (32.81%)	8 (12.5%)	4 (6.25%)	-
Zamzuri et al. (2020) [30]	Peninsular Malaysia	Negeri Sembilan	May-August 2018	*1*. *Macaca fascicularis 2*. *Macaca nemestrina*	212	Real-Time PCR	107 (50.47%)	77 (36.3%)	-	-	-	-	-	-	-	-	-

### Description of included studies

Among all the included studies, one study (14%) was published from year 2000–2010 [18], and six studies (86%) were published from year 2011–2021 [10,26–30]. Five out of seven studies (71%) were conducted in Peninsular Malaysia which had covered the state of Pahang [18,28,29], Johor [29], Perak [29], Selangor + The Federal Territory of Kuala Lumpur which also known as the Klang Valley [10,18,28], Kedah [28], Kelantan [28], Terengganu and Negeri Sembilan [30]. Meanwhile, two out of seven studies (29%) were carried in East Malaysia or more commonly known as Borneo; one was at Sarawak specifically in Kapit District [26] and another one was in Sabah [27].

*Macaca fascicularis* (long-tailed macaque) and *M*. *nemestrina* (pig-tailed Macaque) were known to be the subjects in all seven studies. One of the studies has included *Pongo pygmaeus* (Bornean Orang-Utan) in their study, but the data were excluded due to insufficient details [27]. Meanwhile, another study has detected human plasmodium in macaques in their study, however the data was excluded due to the nature of this article [30]. The sample size of macaques in all studies was varied from a minimum of 41 individuals to a maximum of 415 individuals. Blood samples were collected in all studies for the detection of the *Plasmodium* parasite.

Six out of seven (86%) studies had utilised nested PCR [10,18,26–29]. One out of seven (14%) study has utilised real-time PCR with the use of genus and species-specific primer [30]. Out of six studies that applied nested PCR for the analysis, only one had amplified the CtyB gene from mitochondria DNA (mtDNA) [27], while the rest of the studies (83%) were targeting the 18S ribosomal RNA genes [10,18,26,28,29].

All studies showed positive detection of *Plasmodium* parasites in their subjects. The highest percentage of *Plasmodium* positive subjects is 100% [27], meanwhile, the lowest is 11.6% [28]. Overall, the average *Plasmodium* positive percentage from macaques’ blood samples is 59.98%.

### The prevalence of *Plasmodium* species in macaques

Table 2 shows the prevalence of *Plasmodium* species in macaques. The prevalence of *P*. *knowlesi* infection was shown in all studies (7/7) [10,18, 26–30] while *P*. *cynomolgi*, *P*. *coatneyi*, *P*. *fieldi* and *P*. *inui* prevalence were shown in 4/7 studies [10,26,27,29]. Generally, over the seven studies and five *Plasmodium* species, *P*. *inui* has the highest average prevalence (49.27%) and *P*. *fieldi* has the lowest prevalence average (4.76%). *Plasmodium knowlesi* have almost the same prevalence average (26.86%) as *P*. *coatneyi* (26.82%) with only 0.04% indifference. Meanwhile, the average prevalence of *P*. *cynomolgi* (33.05%) is the second highest among the five *Plasmodium* species. One study had observed quite number of human plasmodia like *P*. *vivax*, *P*. *ovale* and *P*. *malariae* in their macaques’ blood samples but the data was not included [30].

**Table 2:** The *Plasmodium* species prevalence in selected articles.

References (n = 7)	*Plasmodium* Species Prevalence (n = 5)				
	*P*. *knowlesi* (%)	*P*. *cynomolgi* (%)	*P*. *coatneyi* (%)	*P*. *feldi* (%)	*P*. *inui* (%)
Vythilingam et al. (2008) [18]	6.80	-	-	-	-
Lee et al. (2011) [26]	78.00	56.00	66.00	4.00	82.00
Akter et al. (2015) [10]	30.00	25.71	22.85	1.42	32.85
Muehlenbein et al. (2015) [27]	14.63	9.75	4.87	9.75	41.46
Huddin et al. (2019) [28]	11.60	-	-	-	-
Amir et al. (2020) [29]	10.67	40.77	13.59	3.88	40.77
Zamzuri et al. (2020) [30]	36.32	-	-	-	-
**Average**	26.86	33.05	26.82	4.76	49.27

### The prevalence of simian malaria infection type

The prevalence of simian malaria infection type was shown in Table 3. In total, only three studies were included as they provided the number of infection types [10,26,29]. The infection types can classify into mono-infection, dual infection, triple infection, quadruple infection and quintuple infection; these classes were indicated that the individual study subjects (macaques) were infected by one, two, three, four, five different *Plasmodium* species respectively. Overall, dual infection (17.59%) was discovered mainly in an individual macaque and quintuple infection (0.92%) is less likely to occur. However, there were not many differences between prevalence of mono, dual and triple infection with only 0.03%-0.18% differences. All in all, macaques in Malaysia were likely to be infected by either one, two or three of *Plasmodium* in an individual.

**Table 3:** The infection type prevalence in selected articles.

References (n = 3)	Infection Type Prevalence (%)				
	Mono	Dual	Triple	Quadruple	Quintuple
Lee et al. (2011) [26]	9.26	16.67	27.78	38.89	0.92
Akter et al. (2015) [10]	12.86	15.71	17.14	4.28	-
Amir et al. (2020) [29]	30.10	20.39	7.76	3.88	-
**Average**	17.41	17.59	17.56	15.68	0.92

Subsequently, the details of the infection type were breakdown in Table 4. In mono infection macaques were commonly found infected by *P*. *cynomolgi* (9.00%) and *P*. *fieldi* (6.50%) compared to the rest of the simian plasmodium. For dual infection, macaques were often identified with the mix of *P*. *cynomolgi* with *P*. *inui* (10.00%) and the mix of *P*. *knowlesi* with *P*. *inui* (6.0%). The triple infection in macaques were predominantly comprise with the mix of *P*. *knowlesi*, *P*. *cynomolgi* and *P*. *coatneyi* (10.50%). Lastly, for quadruple infection, macaques were found most with the combination of *P*. *knowlesi*, *P*. *cynomolgi*, *P*. *coatneyi* and *P*. *inui* (23.00%).

**Table 4:** The details breakdown of the plasmodia species in each infection type that comprise of mono, dual, triple, quadruple and quintuple infection.

INFECTION TYPE	SPECIES	REFERENCES (n = 3)			TOTAL	
		Lee et al. (2011) [26]	Akter et al. (2015) [10]	Amir et al. (2020) [29]	n	(%)
**Mono**	PK	1	4	0	5	2.50
	PCOT	3	2	3	8	4.00
	PCY	1	2	15	18	9.00
	PF	0	1	12	13	6.50
	PIN	5	0	1	6	3.00
	Sum	10	9	31	50	25.00
**Dual**	PK+PCOT	1	0	1	2	1.00
	PK+PCY	2	1	0	3	1.50
	PK+PFI	1	0	0	1	0.50
	PK+PIN	8	4	0	12	6.00
	PCY+PIN	4	3	13	20	10.00
	PIN+PCOT	2	2	5	9	4.50
	PCY+PCOT	0	1	2	3	1.50
	Sum	18	11	21	50	25.00
**Triple**	PK+PCY+PCT	3	1	0	4	2.00
	PK+PCY+PIN	7	4	6	17	8.50
	PK+PIN+PCT	17	4	0	21	10.50
	PK+PIN+PF	1	0	0	1	0.50
	PCY+PCT+PIN	2	3	1	6	3.00
	PCY+PIN+PF	0	0	1	1	0.50
	Sum	30	12	8	50	25.00
**Quadruple**	PK+PCY+PCT+PIN	41	3	2	46	23.00
	PK+PCT+PF+PIN	1	0	0	1	0.50
	PK+PCY+PF+PIN	0	0	2	2	1.00
		42	3	4	49	24.50
**Quintuple**	PK+PCT+PCY+PF+ PIN	1	0	0	1	0.50
	Sum	1	0	0	1	0.50
	Grand total	101	35	64	200	

PK = *Plasmodium knowlesi*

PCOT = *Plasmodium coatneyi*

PCY = *Plasmodium cynomolgi*

PF = *Plasmodium falciparum*

PIN = *Plasmodium inui*

### The prevalence of malaria infection in different regions of Malaysia

**Fig 2:**
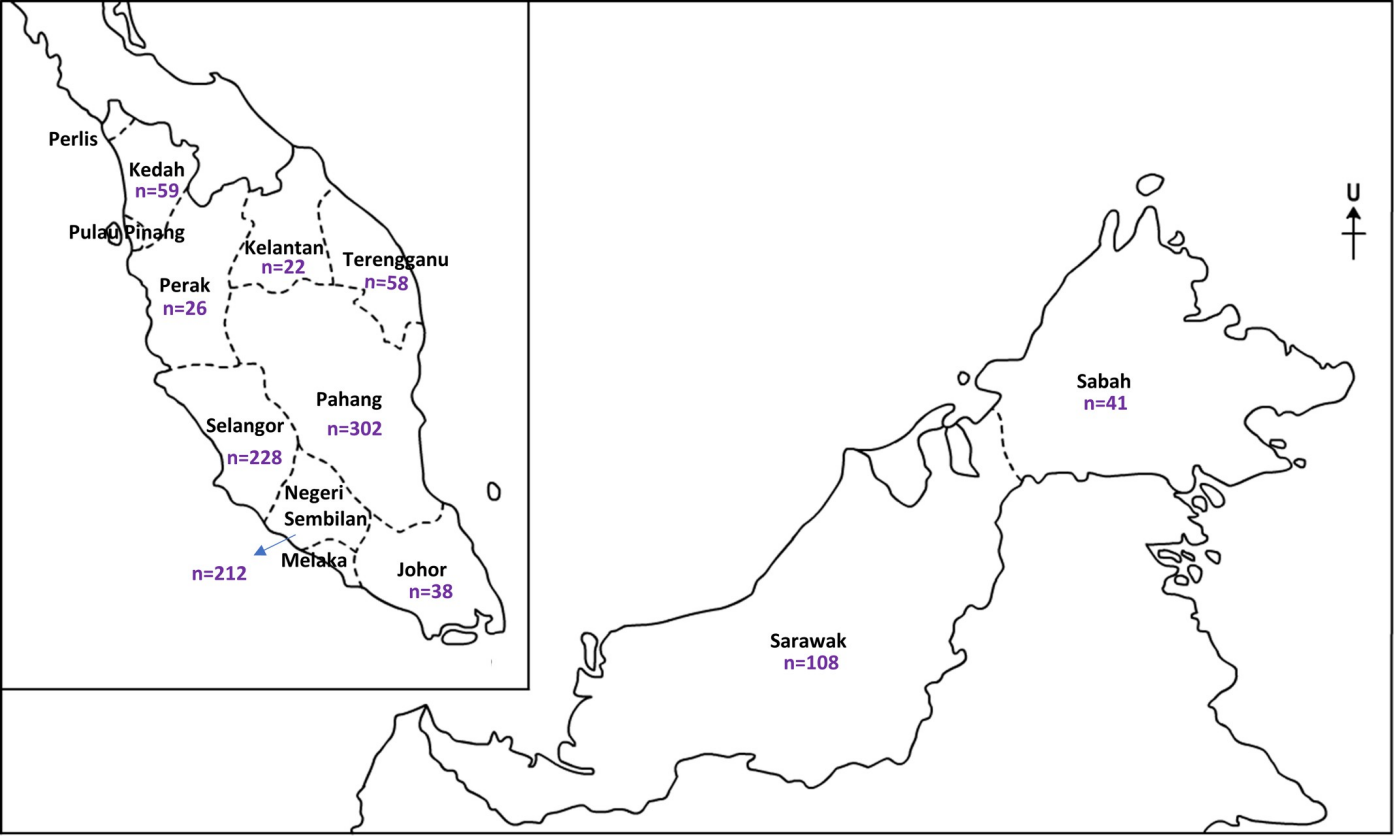
The map of Malaysia (Peninsular and East Malaysia) together with the total number of samples collected in each state (Malaysia map is adapted from https://malaysiageography.blogspot.com/p/peta.html with permission from the owner).

The prevalence of infection in different regions of Malaysia ware shown in Table 5. For over 20 years, five studies had been conducted in Peninsular Malaysia [10,18,28–30] and two studies were conducted in East Malaysia [26,27]. In Peninsular Malaysia, a total of 945 macaques had been sampled for a period of 20 years and 329 of them were positive of malaria parasite. While in East Malaysia, only 149 macaques have been sampled and 142 of them were positive. In general, macaques were sampled in all states of Malaysia except for Perlis, Pulau Pinang and Melaka (Fig 2). Although Peninsular Malaysia has larger macaques sampled, the prevalence of positive malaria parasite was higher in East Malaysia with an average prevalence of 97%.

**Table 5:** The prevalence of *Plasmodium* positive macaques in comparison of geographical location and regions.

Geographical Location (State/Region)	References	Total Macaques (n)	*Plasmodium* positive (n)	Prevalence (%)
Peninsular Malaysia	Vythilingam et al. (2008) [18]	145	75	51.72
	Akter et al. (2015) [10]	70	35	50.00
	Huddin et al. (2019) [28]	415	48	11.60
	Amir et al. (2020) [29]	103	64	62.10
	Zamzuri et al. (2020) [30]	212	107	50.47
	Total	945	329	-
	Average	-	-	45.18
East Malaysia	Lee et al. (2011) [26]	108	101	94.00
	Muehlenbein et al. (2015) [27]	41	41	100.00
	Total	149	142	-
	Average	-	-	97.00
Pahang	Vythilingam et al. (2008) [18]	75	73	97.33
	Huddin et al. (2019) [28]	188	43	22.87
	Amir et al. (2020) [29]	39	35	89.74
	Total	302	151	-
	Average	-	-	69.98
Klang Valley (KL+Selangor)	Vythilingam et al. (2008) [18]	70	2	2.58
	Akter et al. (2015) [10]	70	35	50
	Huddin et al. (2019) [28]	88	0	0
	Total	228	37	-
	Average	-	-	17.52

Next, two of the state in Peninsular Malaysia; Pahang and Klang Valley which made up of Selangor and KL were compared for the prevalence of infection. These two states were selected among all other states because the number of studies has been carried out was more than one; three studies have been conducted in Pahang [18,28,29] and three studies have been conducted in Klang Valley [10,18,28]. In the period of 20 years, 302 macaques were sampled in Pahang and 228 macaques were sampled in Klang Valley and the number of simian malaria positive macaques were 151 and 37 respectively. Pahang has a higher average prevalence (69.98%) compared to Klang Valley (17.52%). Altogether, macaques of East Malaysia have a higher prevalence of *Plasmodium* infection compared to Peninsular Malaysia, and Pahang state in Peninsular Malaysia has a higher prevalence compared to Klang Valley.

## Discussion

The aim of Malaysia to eliminate malaria nationwide by 2020 seems to need to be prolonged. Whilst Malaysia has successfully eliminated the transmission of human malaria [31], a large number of people especially those who live in remote areas are still infected by simian malaria parasite, *P*. *knowlesi* [32–34]. In order to assess the infection risk and properly manage the malaria elimination programme, several epidemiology studies of *Plasmodium* in simian host species have been carried out [10,18,26–30]. This is the first systematic review to determine the prevalence of *Plasmodium* among simian host species in Malaysia that is based on seven full-text English publications dated from 2008–2021.

The main simian malaria host in Malaysia is the long-tailed macaques (*M*. *fascicularis*) and pig-tailed macaques (*M*. *nemestrina*). These macaques are known as the reservoir of five common simian *Plasmodium* species namely *P*. *knowlesi*, *P*. *cynomolgi*, *P*. *coatneyi*, *P*. *fieldi* and *P*. *inui*. This study has found that the most prevalent *Plasmodium* in macaques was *P*. *inui*, followed by *P*. *knowlesi* and *P*. *cynomolgi* and the least prevalent was *P*. *fieldi*. Studies in the Philippines [35] and Thailand [36] also shown that the prevalence of *P*. *inui* was highest among all, but it was lowest in the study conducted in Singapore [37]. The prevalence of *P*. *fieldi* was high in Thailand (30%) [36], Singapore (42%) [37] and the Philippines (41%) [35] but it was the lowest in three of four studies conducted in Malaysia [10,26,27,29]. However, the prevalence of each simian malaria can be affected by the geographical location they were sampled [35]. The reasons for the differences in the number of macaques infected with different or multiple *Plasmodium* parasites at each sampling site were also affected by few variables, which include differences in host genetics, susceptibility of host toward parasite, differences in the vectors between sampling sites and the frequency of sampling on one individual host [38]. There was one study which conducted in Negeri Sembilan shown that macaques were infected with human Plasmodium species; *P*. *vivax*, *P*. *ovale* and *P*. *malariae* [30]. Mix infection of *P*. *knowlesi* with human plasmodium also observed in the sampled subject [30]. Consequently, this finding showed those macaques have the potential of becoming the reservoir not only to simian plasmodium but for human plasmodium as well.

The presence of multiple *Plasmodium* species infection in macaques were detected in Malaysia by the PCR diagnosis method. Though the microscopic examination of Giemsa-stained thick and thin film is the most common malaria diagnostic method. However, it was time-consuming and misdiagnosis of the *Plasmodium* species is common particularly when the parasitaemia is low and inexperience staff [39]. The *P*. *knowlesi* infection may resemble *P*. *malariae* infection and other malaria infections such as *P*. *falciparum* and *P*. *vivax* [40] and *P*. *cynomolgi* would have been misidentified as *P*. *vivax* under the microscope [41]. As these *Plasmodium* species are indistinguishable on regular microscopy, hence upgrading microscopy training will be ineffective. Therefore, *Plasmodium* species detection by using molecular method; PCR-based was adapted. Nested and real-time PCRs are relatively simple, highly specific and have greater sensitivity of detection even when the parasitaemia is low (as low as 2–5 parasites/μl) [39,42]. Furthermore, many PCR assays for malaria diagnosis, including the conventional approach, have been developed using mostly genus- or species-specific sequences of the *Plasmodium* 18S subunit *rRNA* gene [43–47]. When compared to microscopy and rapid diagnostic test (RDT), PCR assays proved to be the most sensitive method in detecting either mono or mixed parasite infections; as both microscopy and RDT have failed to detect the presence of malaria parasites that otherwise PCR could [48]. Since 2010, all *P*. *knowlesi* and *P*. *malariae* for human infection cases in Sabah have been subjected to a PCR test. In 2011, molecular detection was expanded to Sarawak and Peninsular Malaysia [40].

This study found that the mono, dual and triple infections were the most common infections detected in primate hosts in Malaysia and similar observations were reported in Thailand [36], Singapore [37], Cambodia [9] and the Philippines [35]. One study in Malaysia have detected a quintuple infection in one of the macaques [26], the prevalence is relatively low (0.92%) compared to the other types of infection. However, the quintuple infection type was not unusual, this type of infection was also detected in Singapore (2.1%) [37] and the Philippines (8.4%) [35]. This, indicate that an individual macaque can harbour up to five different *Plasmodium* species at once. In Malaysia, the commonly found *Plasmodium* species in mono infection is *P*. *cynomolgi*, and mix of *P*. *cynomolgi* with *P*. *inui* for dual infection; this have found similar in Singapore [37] and Cambodia [9]. Meanwhile the commonly found *Plasmodium* species mixture for triple and quadruple infection in Malaysia macaques are *P*. *knowlesi* + *P*. *cynomolgi* + *P*. *coatneyi* and *P*. *knowlesi* + *P*. *cynomolgi* + *P*. *coatneyi* + *P*. *inui* respectively. The plasmodium mix of triple and quadruple infection were also found in the studies at Singapore and Cambodia but their infection percentage were not as high as Malaysia. The multiple infections in a host are complex, it has been observed that the parasitaemia of *Plasmodium* parasite fluctuates over time within a host [35,38]. Due to this complexity of mixed-species infection, a detection through gold-standard microscopic examination alone was difficult to accurately identify each *Plasmodium* species from one another [36, 38]. Even so, the PCR detection results might not always mirror the total number of definite prevalence of *Plasmodium* present in each individual host [36]. Nonetheless, molecular detection methods like nested and real-time PCRs still remain as the best option to detect *Plasmodium* species in a primate host as the light microscopy detection on Giemsa-stained blood films method are imperfect and often misdiagnosed the actual *Plasmodium* species.

In comparison between the prevalence of simian malaria between regions, this study found the simian malaria prevalence in East Malaysia (97.00%) was higher compared to Peninsular Malaysia (45.18%). The forest cover has played an important role in the prevalence of the disease as studies have found that macaques in rural/forested areas were more infected by *Plasmodium* compared to macaques that live in urban/city areas [35,37]. The forested areas in Peninsular Malaysia as of 2010 was approximately 44% of the land area [39], meanwhile, the Sarawak and Sabah Forest cover was 67% and 64% respectively as per 2018 [40]. In general, East Malaysia has more forest cover compared to Peninsular Malaysia as Peninsular Malaysia is more developed compared to East Malaysia. Besides forest cover, the prevalence of simian malaria is also influenced by forest fragmentation. Peninsular Malaysia has undergone widespread deforestation as a result of increased agricultural activities such as cultivation of oil palm and rubber, timber production and increasing urbanisation since 1970s [49]. These crop plantations increase vectors’ natural breeding sites as well as human exposure to these breeding areas [50]. Unlike *P*. *falciparum* or *P*. *vivax* transmission, people infected by *P*. *knowlesi* are usually exposed to agricultural settings or forests due to the vectors’ exophagic nature [23]. Natural simian malaria infections in humans are more likely to occur when humans disrupt the normal mosquito–macaque circulation chain in the forested area [51]. Furthermore, spatial analysis by Phang et al. [52] showed Lipis district has the highest infection rate when compared with other districts in Pahang as most infected patients worked in the agricultural sector, suggesting frequent exposure to the forest, forest-edge and plantation setting. Thus, this increases the possibility of contact with *Anopheles* mosquitoes as well as macaque populations. To add, *P*. *knowlesi* transmission was also found to be higher in large intact forest patches, pulpwood and oil palm plantations with fragmented landscapes in Sabah [3]. Moreover, human movement into insect vector and wildlife reservoir habitats is a major contributor to the emergence and transmission of zoonotic diseases [3]. Individual risk of infection can be identified by tracking movements of susceptible hosts to high-risk locations, duration spent in each location, time of day that movement occurred and routes [53]. Based on the mosquito and human space use data, more than 90% of infectious bites predicted in areas surrounding households at forest edges in Northern Sabah [3]. Local movement patterns within these communities were largely predictable during peak vector times and could be explained by spatial and environmental factors [3]. Furthermore, a survey within this community found that both men and women were equally exposed to *P*. *knowlesi*, as confirmed by specific antibody responses and data on asymptomatic infections indicates that women have a higher number of non-clinical infections [54,55].

On the other hand, the urban area such as Klang Valley has lower prevalence in simian malaria due to the lack of competent vectors. This is because vectors for simian malaria in Malaysia which is the *Anopheles* mosquitoes of the Leucosphyrus group mainly feed on macaques in the forested areas at higher elevation [23,56,57]. The study also found that macaques sleeping in the forest were more vulnerable to mosquitos’ bite compares to macaques that sleeping by the seaside, this might be due to the preference of mosquitoes to shaded areas in the forest as a breeding ground [35]. Nevertheless, compared to the other primates, macaques’ species are frequent caught in an urban area than in forested areas [30]. Not only do those macaques have the most widespread range of non-human primates, but they are also flexible in their feeding strategies and able to quickly adapt to their changing environment [58–60]. The macaques’ movements ranging from 100 to 600 m per day, and route patterns have been influenced by food distribution, sleep site, predation, and territorial factors. The macaques were in close proximity to human settlements due to the high availability and distribution of food [59]. Therefore, deforestation and changing of land use have always been the main reasons for macaques’ disturbance in human settlement.

When macaques move from the forested area to forest fringe and human settlement, the vectors may have followed their host and adapt to human settlement as well [23]. On that account, there was an increase in the simian malaria infection in humans. Several studies and case reports have shown the infection of other plasmodia besides *P*. *knowlesi* in humans. The first case of a naturally acquired human infection with *P*. *cynomolgi* in Malaysia was a 39-year-old Malay woman from Hulu Terengganu located at the East Coast of Peninsular Malaysia. She lives in a malaria-free modern housing area and had no previous history of malaria. However, there was a small, forested area with infrequent sightings of long-tailed macaques behind her house [61]. Next, six patients in Kapit Hospital were identified with *P*. *cynomolgi* and *P*. *knowlesi* co-infections [62]. A recent study also detected *P*. *cynomolgi* infections among indigenous communities in four states (Perak, Negeri Sembilan, Melaka and Kelantan) in Peninsular Malaysia [63]. *Plasmodium inui* and *P*. *coatneyi* infections were also detected among the indigenous communities in Melaka and Perak state Peninsular Malaysia respectively [63]. In Peninsular Malaysia, specifically in Kelantan, Perak and Pahang states, many of the Orang Asli or known as indigenous people still live in the forested areas. Several studies had shown that indigenous people in Peninsular Malaysia were more prone to malaria infection compare to other races [40].

A recent systematic review also revealed the malaria cases from predominantly causes by human malaria parasite were shifting to simian malaria parasites [31]. Human malaria was responsible for 100% of reported malaria cases in Malaysia between 2000 and 2007, with 18–46 deaths per year. Indigenous malaria cases, on the other hand, decreased from 6071 to 0 (a 100% reduction) between 2008 and 2018, while *P*. *knowlesi* cases rosed from 376 to 4131 cases [64]. Subsequently, a study reported that the human *knowlesi* malaria cases in Malaysia from year 2010–2018 were the highest (18687 cases) compared to the other South East Asia country like Indonesia (481 cases), Brunei (73 cases), Myanmar (49 cases), Thailand (44 cases), Vietnam (38 cases), Laos (10 cases) and Philippines, Singapore, together with Cambodia for less than 10 cases [65]. Hence, these findings indicate that human infections caused by simian malaria are hitherto widely distributed in Malaysia.

While The National Malaria Elimination Strategic Plan that targeted to achieve “malaria-free” status by 2020 were challenged due to the rising case of zoonotic malaria in human and there was no new strategic plan for elimination of zoonotic malaria to date. To reduce simian plasmodium transmission to humans, present malaria prevention initiatives may need to be revised or new approaches created. By the finding of this study shown, the authorities like The Wildlife Department might screen macaques for malaria parasites and restrict their population together with managing the human-macaques conflict. Parallel to this, public health policies not only should focus on raising awareness of *knowlesi* malaria but zoonotic malaria as whole and ensuring that procedures are in place to allow for timely accurate diagnosis and treatment, particularly in high-risk areas. The use of mosquito’s repellent, patch, and net need to encourage mainly for people that live or entering high-risk area.

In the period of 20 years, the number of local simian malaria epidemiology studies were relatively low and among them, only seven articles fulfilled our criteria. The lack of local simian malaria studies might be due to the invasive specimen collection of the blood samples from macaques as this required a proper trapping system and highly trained experts to handle the animals. Therefore, a non-invasive specimen collection method that can substitute for blood sample collection such as urine and faecal sample collection has been introduced by several studies [66,67], revealing that traces of *Plasmodium* can be detected through saliva, urine and a faecal sample of an infected individual. Thus, simian malaria detections from urine and a faecal sample of macaques should be considered for the epidemiology study in the future. Additionally, the future surveys of macaque hosts should be combined with the studies of the Anopheline vectors, because zoonotic malaria does not exist where there are no macaque-mosquito-human interactions.

## Conclusion

*Plasmodium* species that infected primates such as *P*. *knowlesi*, *P*. *cynomolgi*, *P*. *inui* and *P*. *cynomolgi* are emerging causes of malaria in humans. In addition, this study confirmed that these four species together with *P*. *fieldi* were detected among macaques in Malaysia. The prevalence of simian malaria was higher in East Malaysia and the mono, dual and triple infection type is the most common among macaques. More simian malaria epidemiology study among macaques together with *Anopheles* vectors should be carried out in Malaysia to assess the risk of macaques-human infection.

## Supporting information

S1 Data Excel sheet of literature searches from two independent review authors (JS and NAS) used in this systematic review. (XLSX)
